# Pregnancy, pregnancy loss and the risk of diabetes in Chinese women: findings from the China Kadoorie Biobank

**DOI:** 10.1007/s10654-019-00582-7

**Published:** 2019-11-19

**Authors:** Sanne A. E. Peters, Ling Yang, Yu Guo, Yiping Chen, Zheng Bian, Huarong Sun, Yanjie Li, Liming Li, Mark Woodward, Zhengming Chen, China Kadoorie Biobank Collaboration Group, Junshi Chen, Zhengming Chen, Rory Collins, Liming Li, Richard Peto, Daniel Avery, Derrick Bennett, Yumei Chang, Yiping Chen, Zhengming Chen, Robert Clarke, Huaidong Du, Xuejuan Fan, Simon Gilbert, Alex Hacker, Michael Holmes, Andri Iona, Christiana Kartsonaki, Rene Kerosi, Ling Kong, Om Kurmi, Garry Lancaster, Sarah Lewington, John McDonnell, Iona Millwood, Qunhua Nie, Jayakrishnan Radhakrishnan, Sajjad Rafiq, Paul Ryder, Sam Sansome, Dan Schmidt, Paul Sherliker, Rajani Sohoni, Iain Turnbull, Robin Walters, Jenny Wang, Lin Wang, Ling Yang, Xiaoming Yang, Zheng Bian, Ge Chen, Yu Guo, Bingyang Han, Can Hou, Jun Lv, Pei Pei, Shuzhen Qu, Yunlong Tan, Canqing Yu, Huiyan Zhou, Zengchang Pang, Ruqin Gao, Shaojie Wang, Yongmei Liu, Ranran Du, Yajing Zang, Liang Cheng, Xiaocao Tian, Hua Zhang, Silu Lv, Junzheng Wang, Wei Hou, Jiyuan Yin, Ge Jiang, Shumei Liu, Zhigang Pang, Xue Zhou, Liqiu Yang, Hui He, Bo Yu, Yanjie Li, Huaiyi Mu, Qinai Xu, Meiling Dou, Jiaojiao Ren, Jianwei Du, Shanqing Wang, Ximin Hu, Hongmei Wang, Jinyan Chen, Yan Fu, Zhenwang Fu, Xiaohuan Wang, Hua Dong, Min Weng, Xiangyang Zheng, Yijun Li, Huimei Li, Chenglong Li, Ming Wu, Jinyi Zhou, Ran Tao, Jie Yang, Jie Shen, Yihe Hu, Yan Lu, Yan Gao, Liangcai Ma, Renxian Zhou, Aiyu Tang, Shuo Zhang, Jianrong Jin, Zhenzhu Tang, Naying Chen, Ying Huang, Mingqiang Li, Jinhuai Meng, Rong Pan, Qilian Jiang, Jingxin Qing, Weiyuan Zhang, Yun Liu, Liuping Wei, Liyuan Zhou, Ningyu Chen, Jun Yang, Hairong Guan, Xianping Wu, Ningmei Zhang, Xiaofang Chen, Xuefeng Tang, Guojin Luo, Jianguo Li, Xiaofang Chen, Jian Wang, Jiaqiu Liu, Qiang Sun, Pengfei Ge, Xiaolan Ren, Caixia Dong, Hui Zhang, Enke Mao, Xiaoping Wang, Tao Wang, Guohua Liu, Baoyu Zhu, Gang Zhou, Shixian Feng, Liang Chang, Lei Fan, Yulian Gao, Tianyou He, Li Jiang, Huarong Sun, Pan He, Chen Hu, Qiannan Lv, Xukui Zhang, Min Yu, Ruying Hu, Le Fang, Hao Wang, Yijian Qian, Chunmei Wang, Kaixue Xie, Lingli Chen, Yaxing Pan, Dongxia Pan, Yuelong Huang, Biyun Chen, Donghui Jin, Huilin Liu, Zhongxi Fu, Qiaohua Xu, Xin Xu, Youping Xiong, Weifang Jia, Xianzhi Li, Libo Zhang, Zhe Qiu

**Affiliations:** 1 The George Institute for Global Health, University of Oxford, 1st Floor, Hayes House, 75 George Street, Oxford, OX1 2BQ UK; 2 Clinical Trials Service Unit and Epidemiological Studies Unit, University of Oxford, Old Road Campus, Oxford, OX3 7FZ UK; 3 Medical Research Council Population Health Research Unit, University of Oxford, Oxford, UK; 4 Chinese Academy of Medical Sciences, Dong Cheng District, Beijing, China; 5 NCDs Prevention and Control Department, Huixian CDC, Huixian, Henan China; 6 NCDs Prevention and Control Department, Nangang CDC, Nangang District, Haerbin, Heilongjiang China; 7 Department of Epidemiology & Biostatistics, School of Public Health, Peking University Health Science Center, Dongguan, China; 8 The George Institute for Global Health, University of New South Wales, Sydney, Australia; 9 Department of Epidemiology, Johns Hopkins University, Baltimore, MD USA

**Keywords:** Pregnancy, Pregnancy loss, Diabetes, China, Miscarriage, Stillbirth, Abortion, Women

## Abstract

Pregnancy and pregnancy loss may be associated with increased risk of diabetes in later life. However, the evidence is inconsistent and sparse, especially among East Asians where reproductive patterns differ importantly from those in the West. We examined the associations of pregnancy and pregnancy loss (miscarriage, induced abortion, and still birth) with the risk of incident diabetes in later life among Chinese women. In 2004–2008, the nationwide China Kadoorie Biobank recruited 302 669 women aged 30–79 years from 10 (5 urban, 5 rural) diverse localities. During 9.2 years of follow-up, 7780 incident cases of diabetes were recorded among 273,383 women without prior diabetes and cardiovascular disease at baseline. Cox regression yielded multiple-adjusted hazard ratios (HRs) for the risk of diabetes associated with pregnancy and pregnancy loss. Overall, 99% of women had been pregnant, of whom 10%, 53%, and 6% reported having a history of miscarriage, induced abortion, and stillbirth, respectively. Among ever pregnant women, each additional pregnancy was associated with an adjusted HR of 1.04 (95% CI 1.03; 1.06) for diabetes. Compared with those without pregnancy loss, women with a history of pregnancy loss had an adjusted HR of 1.07 (1.02; 1.13) and the HRs increased with increasing number of pregnancy losses, irrespective of the number of livebirths; the adjusted HR was 1.03 (1.00; 1.05) for each additional pregnancy loss. The strength of the relationships differed marginally by type of pregnancy loss. Among Chinese women, a higher number of pregnancies and pregnancy losses were associated with a greater risk of diabetes.

**Electronic supplementary material:**

The online version of this article (10.1007/s10654-019-00582-7) contains supplementary material, which is available to authorized users.

## Introduction

Pregnancy is characterised by major alterations in the metabolic system that may have prolonged effects on maternal risk of developing diabetes [1]. Pregnancy loss is common globally, up to 20% of pregnancies terminated in a miscarriage and an estimated 2.6 million stillbirths in 2015 [2, 3]. Moreover, induced abortions is also prevalent, with 35 per 1000 women aged 15–44 years having induced abortion worldwide in 2010–14 [4].

Women with a history of pregnancy loss are at a higher risk of adverse pregnancy outcomes, including gestational diabetes, in subsequent pregnancies [5–7]. Despite possible shared cardiometabolic aetiology, the long-term effects of pregnancy loss on maternal risk of diabetes have only rarely been studied, and only then in Western populations [8, 9]. It therefore remains uncertain whether pregnancy loss has effects on the risk of new-onset diabetes in later life, and whether different types of pregnancy loss had similar risk, especially outside of Europe and North America. However, women with a history of pregnancy loss are at higher risk of cardiovascular disease in later life, suggesting that pregnancy may have prolonged implications for maternal cardiometabolic risk [10].

Appropriate understanding of the association between pregnancy loss and the risk of diabetes is particularly relevant to China, where the prevalence of diabetes has more than quadrupled in recent decades and is still rising [11, 12]. Moreover, the reproductive patterns of Chinese women have changed significantly over the past several decades, yet still differ importantly from those in the West [13]. To date, no prospective studies in China have assessed the association of pregnancy loss and risk of diabetes later in life. We assessed the relationship between pregnancy and pregnancy loss and the risk of diabetes among 270,000 women from the China Kadoorie Biobank (CKB), a contemporary prospective study across 10 diverse areas in China.

## Methods

### Baseline survey

Detailed information about the study design and procedures of CKB have been reported previously. [14] In brief, between 2004 and 2008, 302 669 women and 210 222 men were recruited from five urban, and five rural, areas of China. At the study assessment clinics, trained health workers administered a laptop-based questionnaire that covered demographic and socioeconomic status, lifestyle factors, personal and family medical history. This included questions on women’s reproductive factors, including number of livebirths, pregnancies, miscarriages, induced abortions, and stillbirths. A range of physical measurements were taken by trained technicians using standard methods. Blood pressure was measured twice on the unclothed right upper arm using an automated A&D UA-779 digital monitor, after participants had rested in the seated position for at least 5 min. If the difference between the two measurements was > 10 mmHg, then a third measurement was taken with the last two readings recorded. The mean values of the two recorded measurements were used for the analyses. Standing height was measured to the nearest 0.1 cm using a stadiometer. Weight was measured to the nearest 0.1 kg using a body composition analyser (TANITA-TBF-300GS; Tanita Corporation), while participants were wearing light clothes (appropriate for the season) and no shoes. BMI was calculated as the weight in kilograms divided by the square of the height in metres (kg/m^2^). A blood sample was collected for long-term storage. Local, national, and international ethical approval was obtained and all study participants provided written informed consent.

### Follow-up for morbidity and mortality

Study participants were followed for cause-specific morbidity and mortality through linkage with regional disease and death registers, and with the national health insurance (HI) system until January 1, 2016. Causes of death are derived from official death certificates and are, where necessary, supplemented by reviews of medical records. Data linkage with HI agencies is carried out every 6 months in each region to retrieve all hospitalised events occurring in that period for study participants. Active follow-up is performed annually to minimise attrition. The primary endpoint in this study was incident diabetes mellitus, as defined by codes E10-E14 in the tenth edition of the International Classification of Diseases (ICD-10). Excluded were individuals with a self-reported history of diabetes or screen-detected diabetes, defined as no self-reported diabetes with a blood glucose level ≥ 7.0 mmol/l and a fasting time > 8 h or a blood glucose level ≥ 11.1 mmol/l and a fasting time < 8 h (nine regions), or as a fasting blood glucose level ≥ 7.0 mmol/l (one region) at baseline. Individuals with a self-reported history of coronary heart disease, stroke, or transient ischemic attack at baseline were also excluded.

### Statistical analyses

Baseline characteristics are presented as means (standard deviation) for continuous variables and as percentages for categorical variables. Cox proportional hazards models were used to estimate hazard ratios (HRs) and 95% confidence intervals (CIs) for incident diabetes by number of pregnancies, miscarriages, induced abortions, and stillbirths. All analyses were stratified by age at risk (5-year strata) and area of residence (10 strata), and adjusted for the highest level of education attained (none, primary, secondary, tertiary or above), household income (< 5000 yuan, 5000–19,999 yuan, ≥ 20,000 yuan), smoking (current, former, never), alcohol use (weekly, occasionally, never), physical activity, systolic blood pressure (SBP), history of hypertension, and body mass index (BMI). Analyses for pregnancy loss were restricted to parous women and were additionally adjusted for the number of live births, and where appropriate, number of miscarriages, induced abortions, and stillbirths. For comparisons involving more than two groups, CIs were estimated using floating absolute risks. [15].

In analyses restricted to women who had ever been pregnant, we estimated the HRs per additional pregnancy. Similarly, the HRs per additional pregnancy loss were estimated among women who had experienced at least one miscarriage, induced abortion, or stillbirth. Subgroup analyses were conducted to obtain the HRs for incident diabetes associated with a history of pregnancy or pregnancy loss by study region, age group, highest level of attained education, BMI category, smoking status, and history of hypertension. Among those with a history of pregnancy or pregnancy loss, we conducted similar subgroup analyses to obtain the HRs associated with each additional pregnancy or pregnancy loss. Separate models were fitted within each subgroup and tests for heterogeneity were used to test for differences between subgroups. Analyses were performed using SAS version 9.3 and R version 3.1.2.

## Results

Of the 273,383 women included in the analyses, the mean baseline age was 50 years and 270,781 (99%) had ever been pregnant. Of these, 10% had a history of miscarriage, 53% had a history of induced abortion, and 7% had a history of stillbirth (Table 1). Women with a history of pregnancy loss were more likely to come from urban areas, to have a higher education level or household income compared with those without such history.

**Table 1:** Baseline characteristics of study participants by number of pregnancy losses

	Total	Never pregnant	Number of pregnancy losses				
			0	1	2	3	≥4
N (% rural)	273,383	2602 (40)	105,273 (70)	84,258 (52)	51,159 (45)	19,139 (46)	10,908 (55)
Age, years	50.1 (10.3)	49.6 (11.5)	50.5 (10.6)	50.0 (10.1)	49.7 (10.0)	50.0 (9.9)	50.5 (10.0)
Education level, %							
Primary or below	56.3	42.6	66.2	52.9	46.6	46.8	51.8
Secondary or above	43.7	57.4	33.8	47.1	53.4	53.2	48.2
Household income, %							
Low	10.2	12.0	13.9	8.0	7.1	7.7	9.9
Middle	49.2	52.3	52.3	45.6	47.1	50.2	54.3
High	40.7	35.7	33.9	46.5	45.8	42.1	35.8
Ever smoker, %	4.9	6.0	4.3	4.5	5.0	6.3	8.6
Physical activity (MET hours/day)	17.7 (11.2, 29.1)	15.0 (9.6, 25.0)	19.1 (11.2, 30.7)	17.6 (11.2, 29.1)	16.8 (10.8, 27.5)	16.1 (10.4, 25.9)	16.1 (10.4, 25.4)
Systolic blood pressure, mmHg	128.7 (21.5)	126.8 (22.6)	130.6 (22.1)	128.2 (21.2)	126.7 (20.6)	126.5 (20.9)	126.7 (20.9)
History of hypertension, %	9.3	8.2	8.8	9.6	9.5	9.7	9.3
Body mass index, kg/m^2^	23.7 (3.4)	23.3 (3.8)	23.5 (3.4)	23.7 (3.4)	23.8 (3.4)	23.9 (3.4)	23.8 (3.4)
Number of pregnancies	3.2 (1.7)	–	2.5 (1.4)	3.0 (1.3)	3.9 (1.2)	4.9 (1.2)	6.7 (1.8)
History of miscarriage, %	9.8	–	–	12.8	14.6	17.7	22.4
History of induced abortion, %	52.7	–	–	79.7	90.7	92.7	92.7
History of stillbirth, %	6.4	–	–	7.6	8.8	11.7	16.0
Number of live births	2.2 (1.3)	–	2.5 (1.4)	2.0 (1.2)	1.9 (1.2)	1.9 (1.2)	2.0 (1.3)

Values are percentages for categorical variables, and means and standard deviations for continuous variables, expect for physical activity where median and 25th and 75th percentile are shown. MET, metabolic equivalent

### Pregnancy and the risk of diabetes

During a median of 9.2 years (Q1: 8.3; Q3: 10.2) of follow-up, 7780 incident cases of diabetes were recorded. There was no difference in the risk of diabetes comparing gravid women to nulligravid women; adjusted HR (95% CI) 0.95 (0.75; 1.20). However, in gravid women, there was a log-linear association between the number of pregnancies and the risk of diabetes (Table 2); the adjusted HRs were 1.00 (0.91; 1.10) for one pregnancy, 1.07 (1.02; 1.12) for two, 1.14 (1.09; 1.18) for three, and 1.22 (1.17; 1.28) for four or more pregnancies. Each additional pregnancy was associated with a 1.04 (1.03; 1.06) increased risk of diabetes. Analyses stratified by age at risk and study area only yielded similar results. Findings were broadly similar across population subgroups (eTable 1 and 2).

**Table 2:** Adjusted hazard ratios (95% confidence intervals) for incident diabetes associated with number of pregnancies and pregnancy losses

	No. events	Model I HR (95% CI)	Model II HR (95% CI)
Pregnancies			
*Ever vs. never*		0.91 (0.72; 1.15)	0.95 (0.75; 1.20)
None	71	1.30 (1.03; 1.64)	1.21 (0.96; 1.53)
1	495	1.00 (0.91; 1.10)	1.00 (0.91; 1.10)
2	1766	1.09 (1.03; 1.14)	1.07 (1.02; 1.12)
3	2117	1.16 (1.12; 1.21)	1.14 (1.09; 1.18)
≥ 4	3331	1.29 (1.24; 1.35)	1.22 (1.17; 1.28)
*Per additional*^*†*^		1.06 (1.04; 1.08)	1.04 (1.03; 1.06)
Pregnancy losses			
*Ever vs. never*^‡^		1.07 (1.02; 1.13)	1.07 (1.02; 1.13)
None	2837	1.00 (0.96; 1.04)	1.00 (0.96; 1.04)
1	2544	1.04 (1.00; 1.08)	1.05 (1.01; 1.09)
2	1463	1.08 (1.03; 1.14)	1.08 (1.02; 1.14)
3	558	1.17 (1.08; 1.27)	1.16 (1.07; 1.26)
≥ 4	307	1.16 (1.04; 1.30)	1.13 (1.01; 1.26)
*Per additional*^*†*^		1.03 (1.01; 1.06)	1.03 (1.00; 1.05)
Miscarriages			
*Ever vs. never*^‡^		1.02 (0.94; 1.11)	1.03 (0.95; 1.12)
None	7050	1.00 (0.97; 1.03)	1.00 (0.97; 1.03)
1	528	1.02 (0.93; 1.11)	1.02 (0.94; 1.11)
≥ 2	131	1.05 (0.89; 1.25)	1.06 (0.90; 1.26)
*Per additional*^*†*^		1.00 (0.89; 1.12)	1.00 (0.89; 1.13)
Induced abortions			
*Ever vs. never*^‡^		1.07 (1.02; 1.12)	1.07 (1.02; 1.13)
None	3554	1.00 (0.96; 1.04)	1.00 (0.96; 1.04)
1	2317	1.05 (1.01; 1.09)	1.06 (1.02; 1.10)
≥ 2	1838	1.10 (1.05; 1.15)	1.09 (1.04; 1.14)
*Per additional*^*†*^		1.04 (1.01; 1.07)	1.02 (0.99; 1.06)
Stillbirths			
*Ever vs. never*^‡^		1.09 (0.99; 1.19)	1.10 (1.00; 1.20)
None	7149	1.00 (0.96; 1.04)	1.00 (0.96; 1.04)
1	432	1.09 (0.99; 1.19)	1.10 (1.00; 1.20)
≥ 2	128	1.08 (0.90; 1.29)	1.09 (0.91; 1.31)
*Per additional*^*†*^		1.00 (0.89; 1.13)	1.00 (0.88; 1.13)

Model I Cox models were stratified by age at risk and study area. Model II: Cox models were stratified by age and study area, and HR were adjusted for level of attained education, household income, smoking status, alcohol use, systolic blood pressure, history of hypertension, physical activity, and body mass index. Model II analyses for pregnancy loss, miscarriage, induced abortion, and stillbirth were additionally adjusted for number of live births, and (where appropriate) number of miscarriages, induced abortions, and stillbirths. †Analyses are restricted to women with at least one pregnancy, pregnancy loss, miscarriage, induced abortion, or stillbirth, respectively. ‡Analyses are restricted to women with at least one pregnancy

### Pregnancy loss and the risk of diabetes

**Fig. 1:**
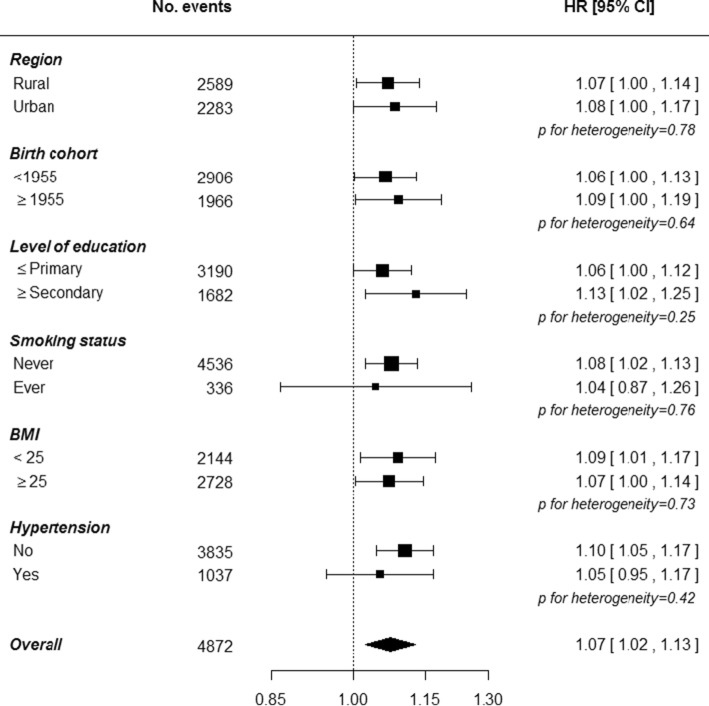
Adjusted hazard ratios for incident diabetes associated with a history pregnancy loss by baseline characteristics. Analyses are stratified by age at risk and study area, and adjusted for level of attained education, household income, smoking status, alcohol use, systolic blood pressure, history of hypertension, physical activity, body mass index, and number of live births. Each closed square represents the risk of diabetes associated with a history of pregnancy loss, with its area inversely proportional to the standard error of the log risk. The diamond indicates the overall risk of diabetes associated with a history of pregnancy loss and its 95% CI. Analyses are among women with at least one pregnancy only

**Fig. 2:**
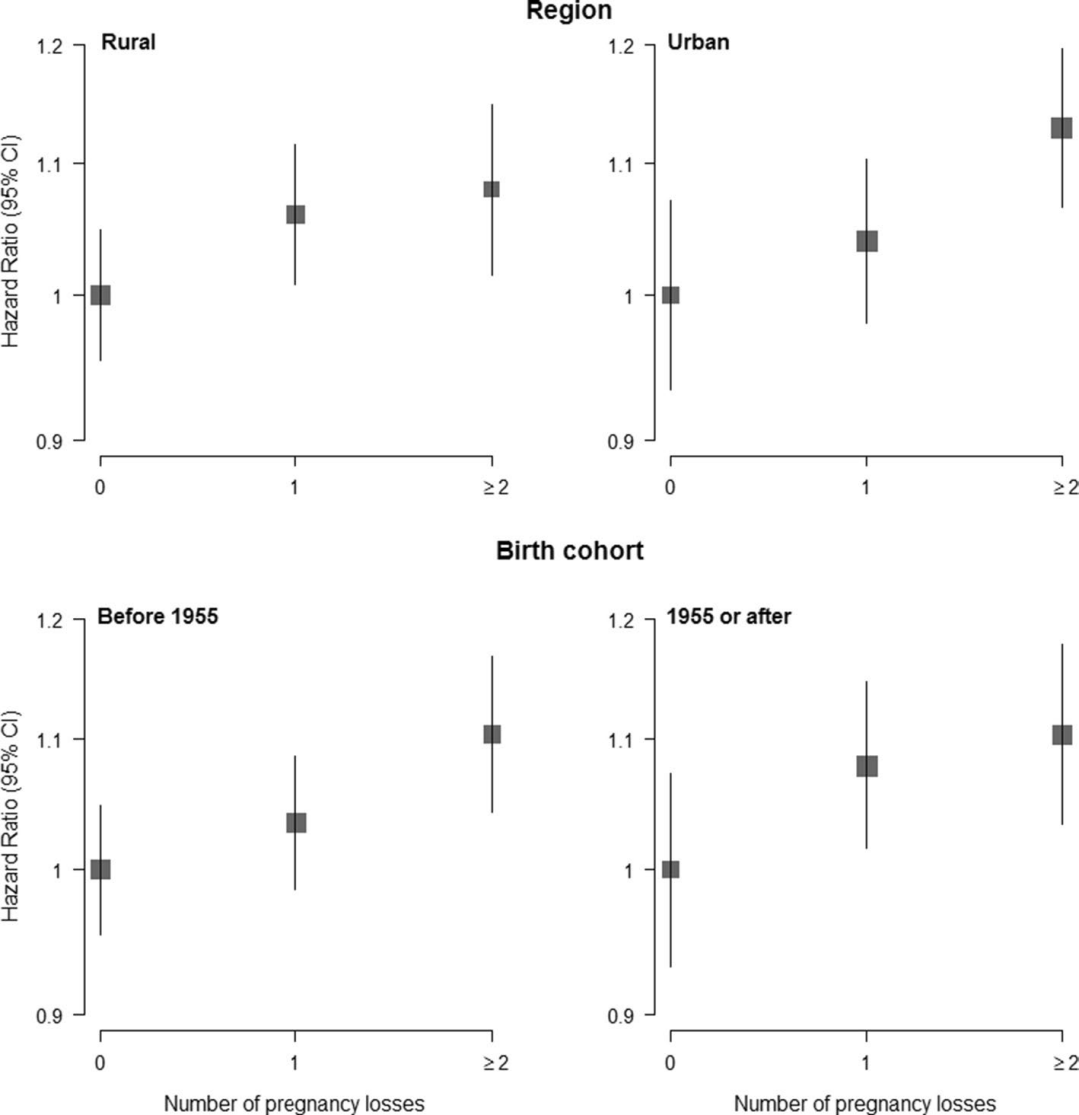
Adjusted hazard ratios (95% confidence intervals) for incident diabetes associated with number of pregnancy losses, by region and birth cohort. Adjustments are as in Fig. 1. The hazard ratios (HRs) are plotted on a floating absolute scale. Each square has an area inversely proportional to the standard error of the log risk. Vertical lines indicate the corresponding 95% confidence intervals (CIs). Analyses are among women with at least one pregnancy only

**Fig. 3:**
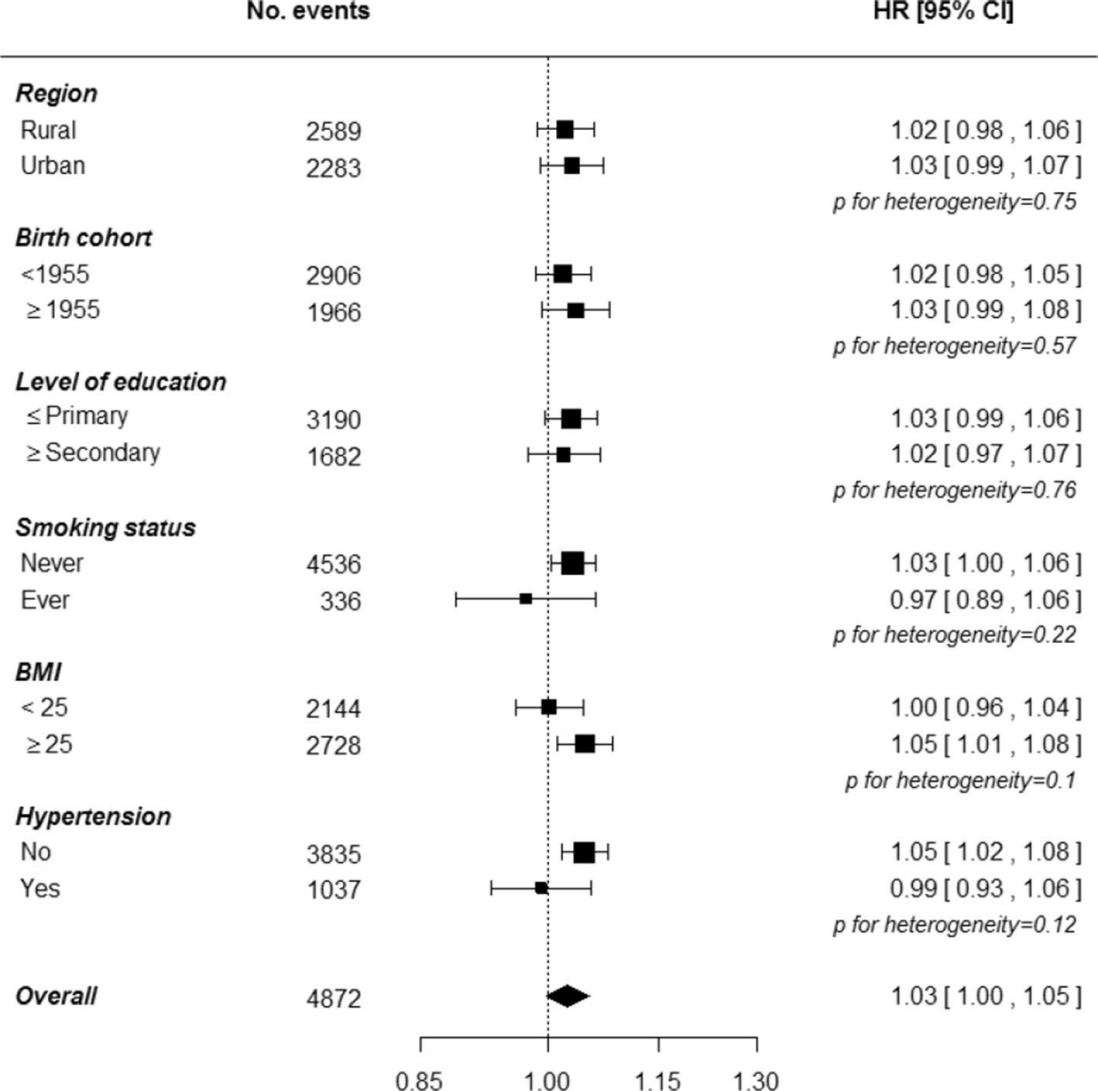
Adjusted hazard ratios for incident diabetes associated with each additional pregnancy loss by baseline characteristics. Adjustments are as in Fig. 1. Each closed square represents the risk of diabetes per additional pregnancy loss, with its area inversely proportional to the standard error of the log risk. The diamond indicates the overall diabetes risk per additional pregnancy loss and its 95% CI. Analyses among women with at least one pregnancy loss only

Compared to women without a history of pregnancy loss, those with a history of pregnancy loss had a 7% higher risk of diabetes (adjusted HR: 1.07 [1.02; 1.13]), with little heterogeneity across population subgroups (Fig. 1). The relationships were directionally similar for different types of pregnancy loss; with adjusted HRs of 1.03 (0.95; 1.12) for a history of miscarriage, 1.07 (1.02; 1.13) for a history of induced abortion, and 1.10 (1.00; 1.20) for a history of stillbirth. The risk of diabetes was higher among those with multiple pregnancy losses; the adjusted HRs were 1.00 (0.96; 1.04) for no pregnancy loss, and 1.05 (1.01; 1.09), 1.08 (1.02; 1.14), 1.16 (1.07; 1.26), and 1.13 (1.01; 1.26) for one, two, three, and four or more pregnancy losses, respectively (Table 2). The results were virtually identical in analyses stratified by age at risk and study area only. This dose–response relationship was consistent between women from rural and urban areas and between those born before 1955 or in 1955 or later (Fig. 2). The HR of diabetes associated with each additional pregnancy loss was 1.03 (1.00; 1.05), with little evidence of differences between subgroups of populations (Fig. 3). Findings were broadly similar in direction and magnitude of the effects for the different types of pregnancy loss, although confidence intervals are wide from the analyses on miscarriage and stillbirth (Table 2 and eTable 1 and 2).

### Joint relationship of the number of livebirths and number of pregnancy losses and the risk of diabetes

**Fig. 4:**
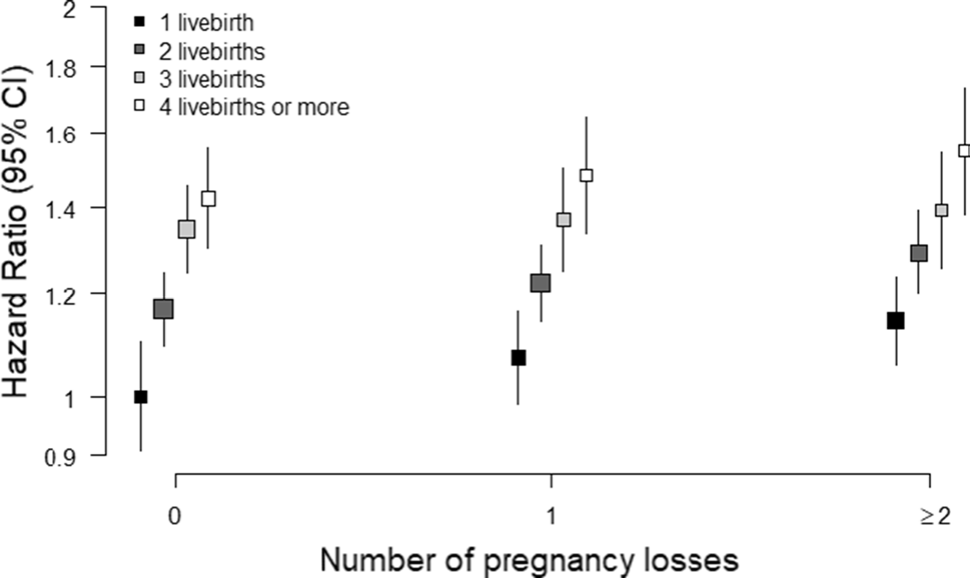
Adjusted hazard ratios (95% confidence intervals) for incident diabetes associated with combinations of the number of livebirths and pregnancy losses. Analyses are stratified by age at risk and study area, and adjusted for level of attained education, household income, smoking status, alcohol use, systolic blood pressure, history of hypertension, physical activity, body mass index. The hazard ratios (HRs) are plotted on a floating absolute scale. Each square has an area inversely proportional to the standard error of the log risk. Vertical lines indicate the corresponding 95% confidence intervals (CIs). Analyses are among women with at least one pregnancy only

There was a graded relationship between the number of pregnancy losses and the risk of diabetes across all groups defined by the number of livebirths (Fig. 4 and eTable 3). Among those with one livebirth, the adjusted HRs of diabetes were 1.00 (0.90; 1.10) those with no pregnancy loss, 1.07 (0.99; 1.15) for one pregnancy loss, and 1.14 (1.07; 1.22) for two or more pregnancy losses. Among those with four livebirths or more, the adjusted HRs of diabetes were 1.42 (1.33; 1.51) those with no pregnancy loss, 1.48 (1.38; 1.59) for one pregnancy loss, and 1.55 (1.44; 1.66) for two or more pregnancy losses.

## Discussion

This large study in China provides the most comprehensive assessment to date of the relationships of pregnancy and pregnancy loss and the risk of diabetes in later life. Among women who had ever been pregnant, each additional pregnancy was associated with a 4% higher risk of diabetes. Moreover, a history of pregnancy loss was associated with a 7% higher risk of diabetes and the relationships became stronger with recurrent pregnancy loss, irrespective of the number of livebirths. These findings were directionally similar for different types of pregnancy loss, were robust to adjustment for potential confounders, and were broadly consistent across major subgroups.

Previous studies on the association between pregnancy and the risk of diabetes have provided conflicting results [8, 16–20]. The US Nurses’ Health study of 114,000 women and 2300 cases of diabetes did find an association between parity and incident diabetes before, but not after, adjustment for BMI [17]. The ARIC study of 7000 women and 750 cases of diabetes also demonstrated that a substantial part of the relationship between parity and diabetes was explained by obesity; after adjustment, only women with five or more live births had a significantly higher risk of diabetes than nulliparous women [20]. Similarly, the Singapore Chinese Health Study among 25,000 Chinese women and 1300 cases of diabetes showed that the association between parity and diabetes was attenuated, but remained significant, after adjustment for BMI and other demographic, lifestyle, and reproductive health factors. [18] In the EPIC-Heidelberg cohort, among 14,000 women and 900 cases of diabetes, each additional pregnancy increased the risk of diabetes before, but not after adjustment for possible confounders. [8] In the present study, which included more cases of diabetes than all previous studies combined, the association between the number of pregnancies and the onset of diabetes was attenuated, yet remained statistically significant, after adjustment for a raft of potential confounders and mediators. However, we recently found that the association between the number of children and the risk of diabetes and cardiovascular disease was largely similar between women and men [21, 22]. Hence, the relationship between pregnancy and the risk of diabetes observed in the present study is likely to be explained by lifestyle and socioeconomic factors related to parenthood and raising offspring, rather than by the direct biological effects of childbearing.

Few studies have examined the relationship between pregnancy loss and new onset of diabetes [8, 9]. The EPIC-Heidelberg study found that a history of miscarriage was associated with a 30% greater risk of diabetes; recurrent miscarriage was associated with a two-fold increased risk of diabetes. [8] No significant associations were found between induced abortion and stillbirth and the risk of diabetes, albeit the small proportion of women who had experienced at least one stillbirth might have hampered the analyses. A study among 3851 women with gestational diabetes and 11,553 women with normal glucose tolerance indicated that stillbirth increased the risk of diabetes by about two-fold, irrespective of gestational diabetes status. [9] This study included a much larger number of well-characterised incident cases of diabetes and provides robust evidence for the implications of different types of pregnancy on the risk of diabetes in a contemporary population of Chinese women.

Pregnancy loss is characterised by multifactorial and polygenic aetiologies that may also be implicated in the onset of diabetes. However, the mechanisms underpinning the association between pregnancy loss and the onset of diabetes in later life are uncertain. We previously reported that women with a history of miscarriage, induced abortion, and stillbirth were at a substantially higher risk of cardiovascular disease, with stronger associations among those with recurrent pregnancy loss. [23] Since diabetes is a strong risk factor for cardiovascular disease, similar pathological mechanisms may be involved. Autoimmune disorders and subclinical inflammatory processes are involved in the pathophysiology of pregnancy loss and there is growing evidence that inflammatory processes also play a role in the development of diabetes. [24–27] Moreover, women with a history of gestational diabetes are at substantially higher risk of type 2 diabetes and previous studies have reported that a history of spontaneous abortion was associated with a higher risk of impaired glucose tolerance or gestational diabetes in later pregnancies. [28] For instance, in a study among 16,000 pregnant women in China, a history of spontaneous abortion was associated with a 50% increased risk of gestational diabetes. [7] A lower socioeconomic status is also associated with a higher risk of spontaneous abortion, indicating that behavioural and environmental exposures more dominant in those with lower socioeconomic status could be involved. [29] In this study, however, the relationships between pregnancy loss and the risk of diabetes were similar in women with different levels of educational achievement. Further studies are needed to elucidate the mechanisms involved by which pregnancy loss might be involved in the pathophysiology of diabetes.

The strengths of the present study include its large sample size, prospective design, and ability to study different types of pregnancy loss simultaneously and to adjust for a range of potential confounders. The generalisability of our findings was enhanced by the inclusion of women from 10 diverse areas in China. While our findings were robust and consistent across a range of analyses, we cannot exclude the possibility that the observed associations have been subject to residual confounding by biomarkers, such as inflammatory variables, and physiological, cultural, or socioeconomic factors underlying the number of pregnancies and/or pregnancy losses not included in our analyses. In particular, information on risk factors before pregnancy or pregnancy-induced conditions, such as polycystic ovary syndrome, preeclampsia, and gestational diabetes, were not available. We were therefore not able to examine to what extent maternal conditions before or during pregnancy have affected the observed relationships between pregnancy loss and diabetes. The number of pregnancies and pregnancy losses were self-reported and may have been subject to recall and reporting bias, resulting in misclassification of the exposure status. However, any misclassification is unlikely to be related to the risk of diabetes in later life, which would have led to conservative estimates.

In summary, we observed graded and positive relationships of pregnancy and pregnancy loss with the risk of diabetes in a contemporary population of Chinese women. Further studies will be needed to examine which physiological, behavioural, and socioeconomic factors might be involved and how these could be mediated to delay or prevent the onset of diabetes among affected women.

## Electronic supplementary material

Below is the link to the electronic supplementary material.

Supplementary material 1 (DOCX 40 kb)
