# Supplementary material for: Pregnancy, pregnancy loss and the risk of diabetes in Chinese women: findings from the China Kadoorie Biobank
Source: Eur J Epidemiol. 2019 Nov 19;35(3):295–303. doi: 10.1007/s10654-019-00582-7 (PMC7154020; doi:10.1007/s10654-019-00582-7)
Supplement: Supplementary file 1 — Supplementary material 1 (DOCX 40 kb) [file 10654_2019_582_MOESM1_ESM.docx]

**Supplementary appendix**

**eTable 1: Adjusted hazard ratios (95% confidence intervals) for incident diabetes associated with a history of pregnancy or pregnancy loss, baseline characteristics**

|  | **Pregnancy** | | **Pregnancy loss** | | **Miscarriages** | | **Induced abortions** | | **Stillbirths** | |
| --- | --- | --- | --- | --- | --- | --- | --- | --- | --- | --- |
|  | **HR (95% CI)** | ***P*** | **HR (95% CI)** | ***P*** | **HR (95% CI)** | ***P*** | **HR (95% CI)** | ***P*** | **HR (95% CI)** | ***P*** |
| ***Region*** |  |  |  |  |  |  |  |  |  |  |
| Rural | 1.01 (0.72; 1.41) |  | 1.07 (1.00; 1.14) |  | 1.02 (0.92; 1.12) |  | 1.05 (0.99; 1.12) |  | 1.13 (1.01; 1.27) |  |
| Urban | 0.90 (0.65; 1.25) | 0.642 | 1.08 (1.00; 1.17) | 0.779 | 1.05 (0.91; 1.21) | 0.721 | 1.10 (1.02; 1.19) | 0.406 | 1.02 (0.86; 1.20) | 0.281 |
| ***Birth cohort*** |  |  |  |  |  |  |  |  |  |  |
| <1955 | 0.96 (0.72; 1.29) |  | 1.06 (1.00; 1.13) |  | 1.02 (0.93; 1.12) |  | 1.06 (1.00; 1.12) |  | 1.09 (0.99; 1.21) |  |
| ≥1955 | 0.90 (0.61; 1.34) | 0.788 | 1.09 (1.00; 1.19) | 0.636 | 1.05 (0.90; 1.22) | 0.746 | 1.09 (1.01; 1.19) | 0.519 | 1.10 (0.89; 1.35) | 0.984 |
| ***Education*** |  |  |  |  |  |  |  |  |  |  |
| Primary or below | 0.96 (0.71; 1.30) |  | 1.06 (1.00; 1.12) |  | 1.01 (0.92; 1.11) |  | 1.05 (0.99; 1.11) |  | 1.12 (1.01; 1.24) |  |
| Secondary or above | 0.93 (0.64; 1.36) | 0.898 | 1.13 (1.02; 1.25) | 0.254 | 1.11 (0.95; 1.31) | 0.299 | 1.12 (1.02; 1.24) | 0.256 | 0.99 (0.78; 1.25) | 0.347 |
| ***Smoking status*** |  |  |  |  |  |  |  |  |  |  |
| Never | 1.05 (0.73; 1.52) |  | 1.08 (1.02; 1.13) |  | 1.04 (0.95; 1.13) |  | 1.08 (1.02; 1.13) |  | 1.09 (0.99; 1.20) |  |
| Ever | 0.79 (0.58; 1.07) | 0.228 | 1.04 (0.87; 1.26) | 0.756 | 0.95 (0.72; 1.25) | 0.548 | 1.00 (0.83; 1.20) | 0.449 | 1.13 (0.82; 1.58) | 0.836 |
| ***BMI*** |  |  |  |  |  |  |  |  |  |  |
| <25 | 0.93 (0.71; 1.21) |  | 1.09 (1.01; 1.17) |  | 0.94 (0.83; 1.06) |  | 1.10 (1.02; 1.18) |  | 1.10 (0.97; 1.26) |  |
| ≥25 | 0.93 (0.56; 1.55) | 0.998 | 1.07 (1.00; 1.14) | 0.728 | 1.11 (0.99; 1.23) | 0.051 | 1.07 (1.00; 1.14) | 0.581 | 1.08 (0.95; 1.23) | 0.806 |
| ***Hypertension*** |  |  |  |  |  |  |  |  |  |  |
| No | 0.95 (0.75; 1.22) |  | 1.10 (1.05; 1.17) |  | 1.03 (0.94; 1.13) |  | 1.08 (1.02; 1.14) |  | 1.11 (1.00; 1.23) |  |
| Yes | 0.80 (0.36; 1.81) | 0.689 | 1.05 (0.95; 1.17) | 0.416 | 1.03 (0.87; 1.23) | 0.977 | 1.04 (0.94; 1.16) | 0.623 | 1.06 (0.89; 1.28) | 0.696 |
|  |  |  |  |  |  |  |  |  |  |  |
| ***Overall*** | 0.95 (0.75; 1.20) |  | 1.07 (1.02; 1.13) |  | 1.03 (0.95; 1.12) |  | 1.07 (1.02; 1.13) |  | 1.10 (1.00; 1.20) |  |

HRs are stratified by age and study area, and adjusted for level of attained education, household income, smoking status, alcohol use, systolic blood pressure, history of hypertension, physical activity, and body mass index. Analyses for pregnancy loss, miscarriage, induced abortion, and stillbirth were additionally adjusted for number of live births, and where appropriate, number of miscarriages, induced abortions, and stillbirths.

Analyses for pregnancy loss, miscarriage, induced abortion, and stillbirth were restricted to women with at least one pregnancy

P represents the p-value for heterogeneity between subgroups

**eTable 2: Adjusted hazard ratios (95% confidence intervals) for incident diabetes associated with each additional pregnancy or pregnancy loss by baseline characteristics**

|  | **Pregnancy** | | **Pregnancy loss** | | **Miscarriages** | | **Induced abortions** | | **Stillbirths** | |
| --- | --- | --- | --- | --- | --- | --- | --- | --- | --- | --- |
|  | **HR (95% CI)** | ***P*** | **HR (95% CI)** | ***P*** | **HR (95% CI)** | ***P*** | **HR (95% CI)** | ***P*** | **HR (95% CI)** | ***P*** |
| ***Region*** |  |  |  |  |  |  |  |  |  |  |
| Rural | 1.04 (1.02; 1.07) |  | 1.02 (0.99; 1.06) |  | 1.01 (0.88; 1.17) |  | 1.03 (0.98; 1.07) |  | 0.99 (0.86; 1.13) |  |
| Urban | 1.04 (1.02; 1.07) | 0.952 | 1.03 (0.99; 1.07) | 0.750 | 0.97 (0.78; 1.22) | 0.760 | 1.02 (0.98; 1.07) | 0.877 | 1.02 (0.78; 1.33) | 0.849 |
| ***Birth cohort*** |  |  |  |  |  |  |  |  |  |  |
| <1955 | 1.04 (1.02; 1.06) |  | 1.02 (0.98; 1.05) |  | 0.98 (0.85; 1.13) |  | 1.02 (0.97; 1.06) |  | 1.03 (0.91; 1.17) |  |
| ≥1955 | 1.05 (1.02; 1.08) | 0.556 | 1.03 (0.99; 1.08) | 0.571 | 1.08 (0.88; 1.33) | 0.430 | 1.03 (0.99; 1.08) | 0.563 | 0.56 (0.28; 1.12) | 0.091 |
| ***Education*** |  |  |  |  |  |  |  |  |  |  |
| Primary or below | 1.04 (1.02; 1.06) |  | 1.03 (0.99; 1.06) |  | 1.02 (0.90; 1.17) |  | 1.03 (0.99; 1.07) |  | 1.00 (0.88; 1.13) |  |
| Secondary or above | 1.05 (1.02; 1.09) | 0.517 | 1.02 (0.97; 1.07) | 0.760 | 0.98 (0.77; 1.24) | 0.731 | 1.02 (0.97; 1.07) | 0.743 | 0.97 (0.56; 1.67) | 0.922 |
| ***Smoking status*** |  |  |  |  |  |  |  |  |  |  |
| Never | 1.05 (1.03; 1.07) |  | 1.03 (1.00; 1.06) |  | 0.99 (0.87; 1.13) |  | 1.03 (1.00; 1.06) |  | 1.01 (0.89; 1.14) |  |
| Ever | 0.99 (0.94; 1.05) | 0.055 | 0.97 (0.89; 1.06) | 0.216 | 0.99 (0.70; 1.40) | 0.988 | 0.98 (0.89; 1.09) | 0.390 | 0.66 (0.30; 1.43) | 0.285 |
| ***BMI*** |  |  |  |  |  |  |  |  |  |  |
| <25 | 1.04 (1.01; 1.06) |  | 1.00 (0.96; 1.04) |  | 0.88 (0.71; 1.08) |  | 1.00 (0.95; 1.05) |  | 1.03 (0.89; 1.21) |  |
| ≥25 | 1.05 (1.03; 1.08) | 0.363 | 1.05 (1.01; 1.08) | 0.103 | 1.08 (0.94; 1.24) | 0.102 | 1.05 (1.01; 1.09) | 0.153 | 0.95 (0.78; 1.16) | 0.502 |
| ***Hypertension*** |  |  |  |  |  |  |  |  |  |  |
| No | 1.06 (1.04; 1.07) |  | 1.05 (1.02; 1.08) |  | 1.04 (0.92; 1.18) |  | 1.04 (1.00; 1.07) |  | 0.99 (0.85; 1.14) |  |
| Yes | 1.05 (1.01; 1.09) | 0.707 | 0.99 (0.93; 1.06) | 0.122 | 0.79 (0.57; 1.10) | 0.125 | 0.97 (0.90; 1.05) | 0.129 | 1.01 (0.81; 1.26) | 0.851 |
|  |  |  |  |  |  |  |  |  |  |  |
| ***Overall*** | 1.04 (1.03; 1.06) |  | 1.03 (1.00; 1.05) |  | 1.00 (0.89; 1.13) |  | 1.02 (0.99; 1.06) |  | 1.00 (0.88; 1.13) |  |

HRs are stratified by age and study area, and adjusted for level of attained education, household income, smoking status, alcohol use, systolic blood pressure, history of hypertension, physical activity, and body mass index. Analyses for pregnancy loss, miscarriage, induced abortion, and stillbirth were additionally adjusted for number of live births, and where appropriate, number of miscarriages, induced abortions, and stillbirths.

Analyses are restricted to women with at least one pregnancy, pregnancy loss, miscarriage, induced abortion, or stillbirth, respectively.

P represents the p-value for heterogeneity between subgroups

| **No. of livebirths** | **No. of pregnancy losses** | **Model I** | **Model II** |
| --- | --- | --- | --- |
| 1 | 0 | 1.00 (0.90; 1.10) | 1.00 (0.90; 1.10) |
| 1 | 1 | 1.07 (0.98; 1.15) | 1.07 (0.99; 1.15) |
| 1 | ≥2 | 1.16 (1.08; 1.24) | 1.14 (1.07; 1.22) |
|  |  |  |  |
| 2 | 0 | 1.27 (1.20; 1.33) | 1.17 (1.10; 1.23) |
| 2 | 1 | 1.32 (1.25; 1.39) | 1.22 (1.15; 1.29) |
| 2 | ≥2 | 1.41 (1.34; 1.49) | 1.29 (1.22; 1.37) |
|  |  |  |  |
| 3 | 0 | 1.46 (1.39; 1.54) | 1.35 (1.27; 1.42) |
| 3 | 1 | 1.50 (1.41; 1.59) | 1.37 (1.28; 1.46) |
| 3 | ≥2 | 1.57 (1.47; 1.68) | 1.39 (1.29; 1.50) |
|  |  |  |  |
| ≥4 | 0 | 1.63 (1.54; 1.71) | 1.42 (1.33; 1.51) |
| ≥4 | 1 | 1.72 (1.62; 1.83) | 1.48 (1.38; 1.59) |
| ≥4 | ≥2 | 1.83 (1.72; 1.95) | 1.55 (1.44; 1.66) |

**eTable 3: Adjusted hazard ratios (95% confidence intervals) for incident diabetes associated with combinations of the number of livebirths and pregnancy losses**

Model I Cox models were stratified by age at risk and study area. Model II: Cox models were stratified by age and study area, and HR were adjusted for level of attained education, household income, smoking status, alcohol use, systolic blood pressure, history of hypertension, physical activity, and body mass index.
